# Aortic Complex Plaque Predicts the Risk of Cryptogenic Ischemic Cerebrovascular Disease Recurrence

**DOI:** 10.14336/AD.2015.0923

**Published:** 2016-03-15

**Authors:** Jing Dong, Xin Ma, Jingyuan Qie, Xunming Ji

**Affiliations:** 1 Department of Neurology and; 2 Department of Neurosurgery, Xuanwu Hospital of the Capital Medical University, Beijing 100053, China

**Keywords:** ischemic cerebrovascular disease, aorta, plaque, CT angiography, recurrence

## Abstract

To evaluate the correlations between aortic complex plaque (ACP) and the recurrence of cryptogenic ischemic cerebrovascular disease (CICVD), and to investigate the clinical significance of ACP in CICVD. Methods CICVD patients (aged 17 to 84 years) admitted into the Department of Neurology, Xuanwu Hospital, from July 2011 to December 2013, were consecutively recruited, and divided into ACP and non-ACP groups according to head and neck computerized tomographic (CT) angiography. Recurrences of cerebral ischemic events (CIEs) were compared between these groups after follow-up. Results A total of 117 patients were enrolled (ACP group: 69, non-ACP group: 48) and followed up for a mean of 9.86 months (range: 3-33). The average age of the ACP group was 62.88 years, with 59.4% older than 60 years; the average age of the non-ACP group was 50.29 years, with 37.5% older than 60 years. At the 6-month follow-up, the recurrence rate of CIEs in the ACP group was significantly higher than that of the non-ACP group (17.0% [7/47] and 0% [0/36], respectively; χ2 = 4.283, P = 0.046). The cumulative recurrence risk for CIEs of the ACP group was significantly higher than for the non-ACP group (P = 0.004). Multivariate Cox survival analysis showed that ACP presence was an independent risk factor for CIE recurrence for CICVD patients (relative risk [RR] = 7.803, 95% confidence interval [CI], 1.827~33.319, P = 0.006). Conclusions ACP increased the recurrence risk of CIE in CICVD, and elderly CICVD patients should receive greater attention regarding the significance of ACP in recurrent CICVD.

Cryptogenic ischemic cerebrovascular disease (CICVD) refers to ischemic stroke with an undetermined origin after intensive workup, which includes cryptogenic ischemic stroke (CIS), and transient ischemic attack (TIA) [1]. CIS accounts for 20%-40% of all ischemic strokes [2-3], and its recurrence risk is not lower than other types of ischemic stroke [4]. Therefore, investigation of the potential causes of CICVD would have great importance in the treatment and secondary prevention of CICVD.

Some previous studies suggested that aortic complex plaque (ACP) was closely correlated with the occurrence and recurrence of CICVD. Aortic plaque thicker than 4 mm or with associated ulceration or mural thrombus was defined as ACP [5]. Amarenco [5] *et al* suggested that the occurrence of ACP in CICVD patients over 60 years of age was higher than in those with clear explanations for an ischemic stroke, but Tullio *et al* thought that there was no significant difference between these two [6]. The French Study of Aortic Plaques (FSAP) [7] reported that ACP was an independent predictor of CICVD recurrence in patients over 60 years of age. While the Stroke Prevention: Assessment of Risk in a Community study (SPARC) [8] followed up community groups and concluded that ACP does not increase the incidence and risk of CICVD. International studies therefore have yet to reach a final conclusion about the correlation between ACP and the occurrence and recurrence of CICVD.

Current methods used for evaluating ACP include transesophageal echocardiography (TEE) [5], computed tomography angiography (CTA) [9], etc. TEE much more clearly demonstrates the morphology and echo intensity of ACP, as well as whether or not there is mural thrombus. However, some research showed that CTA is better than TEE at finding smaller plaques, and more sensitive for ulcer plaque [10], especially in the blind zone of TEE (including the distal segment of the ascending aorta and proximal segment of the aortic arch); the number of plaques found by CTA was about four times that of TEE [11]. Therefore, CTA could to some extent compensate for the deficits of TEE in evaluating aortic plaques.

We used CTA to evaluate ACP, and observed the recurrence rate and related factors influencing cerebrovascular events in CICVD patients with or without ACP, in order to investigate the clinical significance of ACP and related factors in CICVD.

## SUBJECTS AND METHODS

### Study subjects

The CICVD patients admitted into the Department of Neurology, Xuanwu Hospital, from July 19, 2011, to December 31, 2013, were consecutively recruited; the study subjects were required to meet the inclusion criteria, but were not excluded by the exclusion criteria. This study was approved by the ethics committee of Xuanwu Hospital, and all subjects gave informed consent.

Inclusion criteria: (1) at least 16 years old; (2) within 60 days after stroke onset; (3) conscious, able to cooperate with testing, and not allergic to contrast agent (iopromide); (4) cerebral infarction or TIA confirmed by magnetic resonance imaging (MRI), meets the new TOAST (Trial of ORG 10172 in Acute Stroke Treatment) classification criteria, and has the cryptogenic features of the SUDu subtype (Stroke of Undetermined Etiology of uncertain determination) that could not be explained after routine tests [1].

Exclusion criteria: (1) hemorrhagic stroke; (2) routine tests determined a reason for ischemic stroke or TIA; (3) with severe hepatonephric or cardiopulmonary dysfunction, hematologic disorders, or malignancy; (4) with a history of fibrinolytic therapy.

### Baseline information

(1) General information and blood tests: the age, sex, and blood pressure of all patients were recorded; Body mass index, routine blood tests, fasting glucose, triglycerides, total cholesterol, low-density lipoprotein (LDL) cholesterol, high-density lipoprotein (HDL) cholesterol, apolipoprotein A1, apolipoprotein B, glycosylated hemoglobin, homocysteine, plasma fibrinogen, immune parameters (including autoimmune antibodies), and anticardiolipin antibody were evaluated within 24 h after admission.
(2) Common risk factors for ICVD: any history of hypertension, diabetes mellitus, hyperlipidemia, heart disease, ICVD, smoking, or drinking, as well as a family history of ICVD, were determined for all subjects.
(3) All subjects underwent carotid ultrasound and transcranial Doppler ultrasound to determine the presence of arterial stenosis or carotid artery plaque; ECG and echocardiography determined whether there were arrhythmias, abnormal heart structure or dysfunction.

All subjects underwent head and neck CTA to determine the status of blood vessels, and to evaluate ACP.

### Detection of ACP

GE LightSpeed 64-slice spiral CT (General Electric Company) was used, with evaluation from the aortic arch to the top of the head, using a helical scanning mode. A CT-specific high-pressure syringe was used to bolus-inject 60 ml of iopromide via a cubital vein, with a flow rate of 4 ml/s (370 mg iopromide/ml). The smart pre-scanning mode was applied, with monitoring commencing 8 s after the injection and scanning when the contrast agent concentration reached a peak inside the target vessel. The specific scan parameters were as follows: tube voltage 140 kV, tube current 575 mA, pitch 0.516, and layer thickness 0.625 mm. The examination was performed by the physicians in the Department of Radiology. GE AW4.2 workstation (General Electric Company) was used to obtain transverse scan images of the thoracic aorta (including ascending aorta, aortic arch, and descending thoracic aorta); the window width and level were adjusted (the window width was 155% of the average lumen density, and the window level was 65% of the average lumen density); two trained resident doctors measured the thickness of aortic plaque (the plaque thickness was defined as the distance from the highest point of the plaque perpendicular to the wall of the outer membrane of the aorta); the average value was calculated after multiple measurements, plaque morphology and the presence of mural thrombus were also evaluated.

### Evaluation criteria for ACP

**Figure 1.**
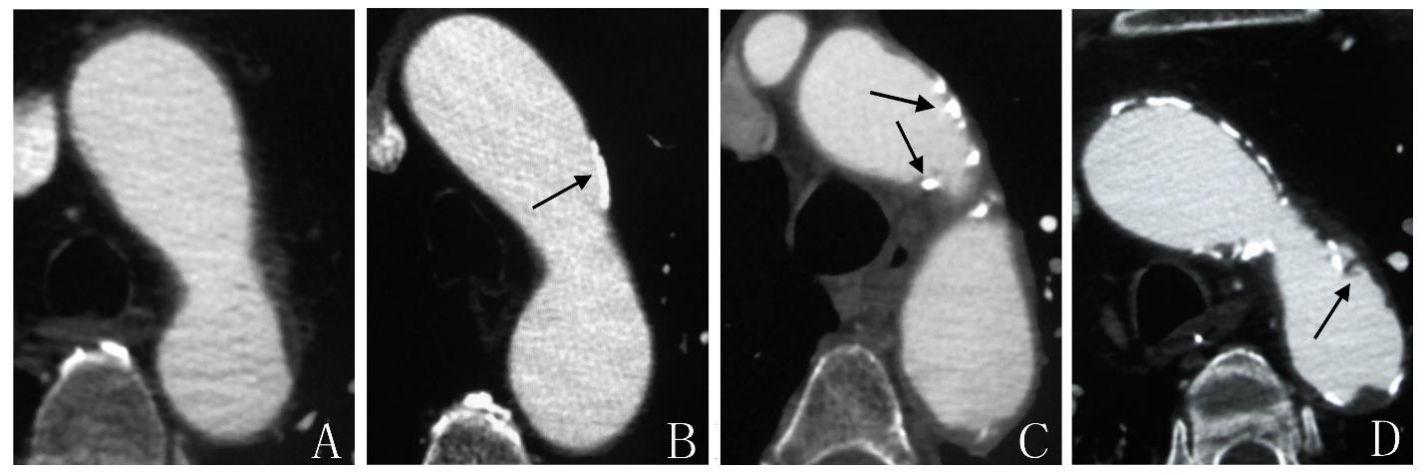
**Axial CTA images of aortic plaques in four grades**. **A)** No plaque at the aortic arch. **B)** a calcified plaque at the lateral wall of aortic arch with thickness less than 4 mm. **C)** Multiple mixed plaques at the aortic arch and descending aorta which are thicker than 4 mm. **D)** ulcer plaque at the lateral wall of aortic arch (black arrows indicate plaques).

**Figure 2.**
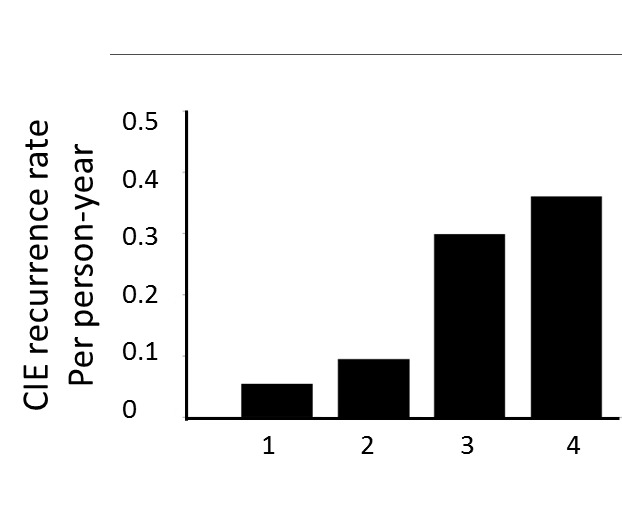
**The link between plaque grade and recurrence rate of CIEs**. The recurrence rate of CIEs per person-year increased with the grade of plaque, the trend appears to increase most sharply when plaques developed into grade 3. CIEs: Cerebral ischemic events.

**Figure 3.**
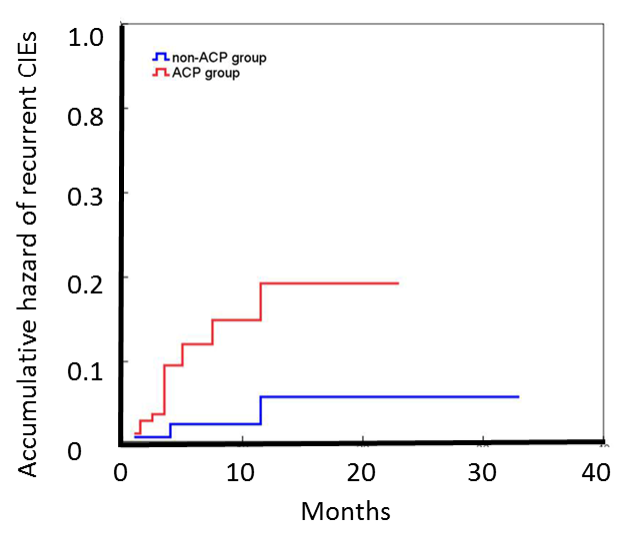
**Accumulative risk of recurrent CIEs for ACP group vs non-ACP group**. Kaplan-Meier analysis for cumulative risk of recurrent CIEs: The cumulative risk of recurrent CIEs in patients with ACP was significantly different from patients without ACP. The *P* value of the Breslow test and log-rank test for the difference were 0.004 and 0.007 respectivel. CIEs: Cerebral ischemic events; ACP: Aortic complex plaque.

Evaluation and classification of aortic atheroma were performed according to the maximum thickness of aortic plaque, ulceration, and mural thrombus; irregular thickening of the aortic wall ≥2mm was collectively defined as aortic plaque [12]; plaque thickness ≥4 mm or associated ulcer or mural thrombus was defined as ACP [7]; maximum defect depth on the plaque surface ≥2 mm was defined as ulcer plaque [12]. All plaques were classified into four grades: 1) no plaque (Figure 1A); 2) plaque with thickness between 2 mm and 4 mm (Figure 1B); 3) plaque thicker than 4 mm (Figure 1C); 4) ulcer plaque irrespective of the thickness (Figure 1D). Grade 1 and 2 are non-ACP plaques, while grade 3 and 4 are ACPs.

### Grouping of study subjects

The study subjects were divided into ACP and non-ACP groups (including cases without aortic atheroma) according to their test results.

### Treatments

All patients were given antiplatelet therapy for secondary prevention; patients with head and neck atherosclerotic changes or nonqualified LDL >70 mg/dl) were given statin therapy; patients with active arterial thrombosis were given anticoagulation therapy.

### Follow-up study

This was a prospective cohort study, using regular inpatient or telephone follow-up to trace the patients after discharge. Follow-up was performed once every three months to determine whether the patients had a recurrence of CIE based on clinical manifestations and the data obtained at follow-up; CIEs that still lacked a known cause were recorded as recurrences. Meanwhile, time of recurrence and other relevant information about CIEs were also recorded. Follow-up ended on March 31, 2014, and was completed by two trained resident physicians.

### Statistical analysis

SPSS 16.0 statistical analysis software (SPSS, Inc.; Chicago, IL, USA) was used. The normally distributed data were expressed as mean ± standard deviation (x± s), and independent sample t-testing was performed; non-normally distributed data were expressed as M (Q25, Q75), and rank testing was performed. Comparisons among the counted data used a single factor χ2 test, and the comparison of recurrence rates used a χ2 test. The Kaplan-Meier method was used to construct a recurrence curve, and the Breslow test and log-rank test were performed to compare recurrences between the two groups. Cox regression was used for multivariate analysis, and the risk ratio was calculated, with the test level set as α = 0.05; P < 0.05 was considered to be a statistically significant.

## RESULTS

Among 135 CICVD patients who met the inclusion criteria, 121 completed head and neck CTA; among these 121 patients, 4 (3.3%) were lost to follow-up (2 had canceled phone numbers, and 2 could not be contacted). Finally, a total of 117 subjects were enrolled, including 69 in the ACP group and 48 in the non-ACP group. Follow-up period ranged from 3-33 months, the median follow-up time was 8 months, with a mean of 9.86 ± 6.06 months.

**Table 1:** Baseline characteristics of ACP group vs non-ACP group

Characteristics	ACP group (n=69)	non-ACP group (n=48)	Value	P
Male, n, (%)*	55 (88.7)	39 (81.3)	0.042	1.000
Age(years)	62.88±12.22	50.29±15.00	4.990	0.000†
HICVD, n, (%)*	14 (20.3)	8 (16.7)	0.243	0.810
Hypertension, n, (%)*	57 (84.1)	37 (77.1)	0.547	0.486
Diabetes mellitus, n, (%)*	15 (21.7)	7 (14.6)	0.949	0.471
Hyperlipidemia, n, (%)*	11 (15.9)	10 (20.8)	0.460	0.625
Choronary heart diseases, n, (%)*	12 (17.4)	4 (8.3)	1.967	0.184
Smoking, n, (%)*	40 (58.0)	26 (54.2)	0.167	0.708
Drinking, n, (%)*	32 (46.4)	16 (33.3)	1.991	0.184
Body mass index (kg/m2)	25.37±2.89	24.76±2.87	0.865	0.390
Fast glucose (mmol/L)	5.67±1.88	5.03±0.87	2.187	0.031†
Triglycerides (mmol/L)	1.64±0.80	1.83±0.99	-1.131	0.260
Total cholesterol (mmol/L)	4.14±1.01	4.20±1.01	-0.334	0.739
LDL-C (mmol/L)	2.56±0.89	2.46±0.84	0.605	0.546
HDL-C (mmol/L)	1.17±0.28	1.23±0.30	-1.090	0.278
Apolipoprotein A1 (g/L)	1.28±0.28	1.23±0.17	1.023	0.309
Apolipoprotein B (g/L)	0.80±0.23	0.76±0.22	0.881	0.380
Fibrinogen (g/L)	3.51±0.76	3.14±0.92	2.339	0.021†
Homocystein (mmol/L,M (Q25,Q75))	21.92 (12.05,23.50)	18.24 (10.10,17.00)	-2.540	0.011†

* Percentage calculated on the total number of patients;

† *P*<0.05 indicates statistical significance. ACP: Aortic complex plaque. HICVD: History of ischemic cerebrovascular diseases.

### Comparison of baseline data

The comparison of baseline data between the two groups revealed that the ACP group exhibited higher age, fasting blood glucose level, plasma fibrinogen level, and homocysteine level compared to the non-ACP group, and the differences were statistically significant; other baseline data showed no significant difference (Table 1). In addition, immunohistochemical indicators including autoimmune antibodies and anticardiolipin antibody in all subjects showed no obvious abnormality. The average age of the ACP group was 62.88 years, with 59.4% older than 60 years; the average age of the non-ACP group was 50.29 years, with 37.5% older than 60 years.

In accordance with the diagnostic criteria of the SUDu subtype, all enrolled subjects underwent carotid ultrasound, transcranial Doppler ultrasound, and CTA examination, to exclude vulnerable plaques, moderate to severe stenosis or occlusion of cranial and carotid arteries which are responsible for stroke.

### Recurrence of CIEs

The ACP group had 16 cases of recurrent CIEs (7 cases of cerebral infarction and 9 cases of TIA), while the non-ACP group had 3 cases (2 cases of cerebral infarction and 1 case of TIA). Ulcer plaques were detected in 43.5% cases of ACP group (30/69); the proportion of recurrent CIEs with associated ulcer plaque was 30% (9/30), while that without ulcer plaque was 17.9% (7/39).

1) Comparison of recurrence rate of CIEs between the two groups. All enrolled patients were followed up for more than three months, with 35 followed up for ≥12 months, and 83 for ≥6 months (47 ACP group cases and 36 non-ACP group cases). As shown in Table 2, at 3-month follow-up, the ACP group exhibited a higher recurrence rate than the non-ACP group, but the difference was not significant (P = 0.254). At 6-month follow-up, the ACP group exhibited a statistically significantly higher recurrence rate than the non-ACP group (P = 0.046).
2) The link between plaque grade and recurrence rate of CIEs. All patients were classified according to the grade of plaques. The CIE recurrent rate per person-year was 5.53%, 9.52%, 29.9% and 36.1% for grade 1 to 4 respectively. As shown in Figure 2, the recurrence rate of CIEs per person-year increased with the grade of plaque, the trend appears to increase most rapidly when plaques developed into ACPs. Based on this, the cumulative risk of recurrence was compared between ACP group and non-ACP group. The Kaplan-Meier method was used to draw a recurrence curve, and the Breslow test and log-rank test were used to perform intergroup comparisons for recurrences. As shown in Figure 3, the cumulative risk of recurrence for CIEs in the ACP group was significantly higher than in the non-ACP group (Breslow test, P = 0.004, log-rank test, P=0.007).
3) Correlations between ACP and the recurrence of CIEs in CICVD patients: multivariate Cox survival analysis was used to correct the baseline data of the two groups (age, fasting blood glucose, plasma fibrinogen, and homocysteine) and other common risk factors for ICVD (triglycerides, total cholesterol, LDL, history of hypertension, ICVD, smoking, and drinking); the results revealed that the presence of complex aortic plaques was an independent predictor of recurrent cerebral ischemic events other than fibrinogen level and history of ischemic cerebrovascular diseases (RR=7.803, 95% CI, 1.827~33.319, *P* = 0.006) (Table 3 and 4).

**Table 2:** Recurrence rate of CIEs for ACP group vs non-ACP group followed over 6 months (case, %)

Recurrence time	ACP group (n=47)	non-ACP group (n=36)	χ^2^	P
3 months	3(6.4)	0	2.384	0.254
6 months	7(17.0)	0	4.283	0.046

CIEs: Cerebral ischemic events; ACP: Aortic complex plaque.

**Table 3:** Independent predictors for cerebral ischemic events in univariable COX regression model

Variables	Relative risk	95% CI	P value
ACP group	4.651	1.342-16.128	0.015
Fibrinogen	2.171	1.273-3.704	0.004
HICVD	3.315	1.281-8.580	0.013

CI: Confidence interval; HICVD: History of ischemic cerebrovascular diseases.

**Table 4:** Independent predictors for cerebral ischemic events in multivariable COX regression model

Variables	Relative risk	95% CI	P value
ACP group	7.803	1.827-33.319	0.006
Fibrinogen	2.258	1.161-4.389	0.016
HICVD	3.987	1.216-13.069	0.022

CI: confidence interval; HICVD: history of ischemic cerebrovascular diseases.

## DISCUSSION

We observed and analyzed recurrences of CIE in CICVD patients with or without associated ACP; the results showed that the presence of ACP increased the recurrence rate of CIE in CICVD patients, and was an independent risk factor.

The FSAP study [7] performed a 2.4-year follow-up of CIS patients, and found that the recurrence rate of CIE in CIS patients with ACP was significantly increased (RR = 5.2, 95% CI, 1.7~15.6, P = 0.0042), consistent with our findings. The FSAP study subjects were those with unclear TOAST-type ischemic stroke, who might have coexisting contributory head and neck arterial stenosis, atrial fibrillation, or other causes; most other studies also set unclear TOAST-type as an inclusion criterion [13], while our study used one new TOAST-type, namely the SUDu subtype, to strictly screen CICVD patients for enrollment; this excluded the impact of various coexisting reasons or inadequate evaluation of the results, to better reveal the relationships between ACP and CICVD.

Aortic plaque might just be one of the presentations in atherosclerosis, and might not necessarily lead to CIE [8]. When the thickness and instability (such as associated ulcer and mural thrombus) of aortic plaque increased and formed ACP, it might increase the possibility of brain and multi-site embolic events. Previous studies detected the presence of ulcer plaque and mural thrombus in ACP through autopsy [14], TEE [7], transthoracic echocardiography (TTE) [15] and CTA [9]; monitoring revealed that ACP might increase intracranial microembolic signals [16], suggesting that the mechanism of ACP-induced CIE was cerebral embolism, in which the surface plaque or mural thrombus broke off and entered the cerebral artery with blood flow, thus causing a blockage of distal vessels and cerebral ischemic changes. In this study, the ACP group exhibited 43.5% associated ulcer plaque, while the recurrence rate of CIEs was higher than in those without ulcer plaque (30% vs. 17.9%), further suggesting that the instability of ACP was one of the important factors contributing to the recurrence of CIEs. Therefore, in clinical work, the morphology and properties of ACP need to be determined, which would enable more effective treatment and secondary prevention in CICVD patients.

ACP was not rare in patients with ischemic stroke [5,7], especially in older CICVD patients. Amarenco [14] *et al* found through autopsy that aortic ulcer plaque was only seen in patients with cerebral infarction who were over 60 years of age, with incidence also increasing with age; the incidence of ACP in CICVD patients over 60 was higher than in those with a clear reason for ischemic stroke [5]. Gu [17] *et al* also found that the incidence of ACP in CIS patients older than 55 years of age was 60%, but only 18% in those less than 55. In this study, the ACP group comprised 59.4% of patients older than 60 years, which was higher than the non-ACP group (37.5%), suggesting that ACP was much more closely correlated with elderly CICVD patients; thus, in clinical practice, middle-aged and older CICVD patients should be more concerned about ACP.

At 3-month follow-up, the cumulative recurrence rate of CIE in the ACP group exhibited an increasing trend compared to the non-ACP group, but the difference was not statistically significant (P = 0.254); at 6-month follow up, the cumulative recurrence rate of CIE in the ACP group was significantly higher than the non-ACP group (P = 0.046), suggesting that CICVD patients with ACP might have a risk of recurrence within 6 months; this risk would further increase with time, and therefore patients with ACP should receive effective early intervention, as well as long-term secondary prevention. Because the follow-up time in this study was short, the long-term impact of ACP on the recurrence of CIE in CICVD patients requires further study.

This study had some limitations. Because we screened the CICVD patients according to the new TOAST criteria, the inclusion criteria were strict, and the number of enrolled patients was therefore relatively small; in addition, the follow-up time was relatively short. Furthermore, this study did not conduct TEE on all study subjects, or exclude the influence of other potential causes, such as abnormal cardiac channels, etc. In future, the sample size should be expanded, the follow-up period should be extended, and certain tests should be reinforced to further clarify the clinical significance of ACP in CICVD.
